# Compensatory beliefs in the internet gratification behavior: A study of game-based assessment

**DOI:** 10.3389/fpubh.2023.997108

**Published:** 2023-01-24

**Authors:** Bin Yin, Yong Shen

**Affiliations:** ^1^Laboratory for Learning and Behavioral Sciences, School of Psychology, Fujian Normal University, Fuzhou, Fujian, China; ^2^Department of Applied Psychology, School of Psychology, Fujian Normal University, Fuzhou, Fujian, China

**Keywords:** compensatory beliefs, internet gratification behavior, game-based assessment, preventative mental health care, population sampling, interactive narratives

## Abstract

Internet gratification behaviors (IGB) may lead to sub-optimal performance in schools and workplace as well as mental health problems such as Internet addiction. The present research examines this phenomenon, focusing on the compensatory belief (CB)—a belief that the negative impact of a certain behavior can be compensated or neutralized by another positive behavior—as a potential psychological mechanism for IGB. An interactive-narrative-style game-based assessment was designed and responses from a random-sampled population of 1,298 participants including college students and organizational employees were collected online. It was found that around 40% of college students and organizational employees would activate some kind of compensatory beliefs when facing with the internet temptation. Those who failed to perform compensatory behaviors afterwards were more likely to regret than those who were able to perform them, which was consistent with the prediction of the CB theory. This study expands the applicability of the CB theory to the field of internet addiction, enriches the understanding of the psychological mechanisms of internet addiction, and suggests that the interactive-narrative-style game-based assessment may be a practical method to study the CB.

## 1. Introduction

Many previous studies focused on Internet addiction. However, before one can be diagnosed with Internet addiction, there may exist a behavioral state that did not reach the pathological use of the Internet, but were deviant from a normal state of internet usage–we call it the Internet gratification state. The Internet gratification (IG) refers to various items on the Internet that can give individuals satisfaction (1). According to Swanson (2), all sorts of gratifications can be divided into content gratifications and process gratifications, as are in the IG. When referring to the IG, content gratifications refer to the satisfaction of finally establishing a connection with the real world, such as finding a job or asking for delivery through the use of the Internet; and process gratifications refers to the process of using the Internet to entertain, such as watching dramas, playing games, etc. Studies have shown that seeking process gratifications through the Internet is significantly associated with individual Internet addiction (1). What's more, unbalanced engagement in the IG may have negative effects on individual wellbeing and lead to psychological problems such as the gaming disorder (3).

The reason why people are so keen on the IG may be explained by the compensatory belief theory. Compensatory beliefs (CB) refer to the individual's belief that the negative impact of a certain behavior can be compensated or neutralized by another positive behavior (4, 5). For example, someone enjoys eating snacks, but he or she knows that it is unhealthy for the body. Therefore, he or she is most likely to activate a kind of compensatory belief (say “anyway I am going to lose weight by doing more exercises later”) and adjust his or her cognitive strategies to accept the temptation (i.e., snacking) without having to endure cognitive conflicts, or cognitive dissonance. In accordance with previous research (6), cognitive dissonance is “the perception of a discrepancy among cognitions generating a negative intra-personal state.” In the CB theory, cognitive dissonance arises from the inconsistency between the individual's desire to be instantly gratified and the long-term health goals (7), and the role of CB is as a strategy to reduce this imbalance within the individual (5). In other words, when facing with the temptation of IG, people can have three choices under the recognition of the existence of cognitive dissonance: (1) Changing their beliefs about IG by reappraising the adverse outcomes of IG (similar to denial) and thus directly accept the temptation; (2) Changing their behavior toward IG by resisting the temptation with resoluteness; (3) Mitigating the conflicts by activating the CB and then accept the temptation with ease in the hope that the compensatory behaviors would ultimately diminish the negative impact of IG [which is different from the theory of Temporal Discounting in which people evaluate their economic gain or loss between now and the future (8–12)]. However, research shows that not everyone does perform compensatory behaviors after accepting the temptation, leaving people with regrets and a feeling of low self-efficacy (13). The CB theory thus emphasizes the role of self-efficacy after activating the CB. If the individual's original self-efficacy is low, he or she will not care whether the compensatory behavior will be performed, and is unlikely to implement it, entering a self-defeating cycle, which may lead to the pathological state of Internet addiction; on the contrary, once the compensatory behavior is efficaciously performed, the individual's feeling of self-efficacy will increase, prompting the individual to quit the malicious cycle and maintain a healthy state of internet usage.

**Figure 1:**
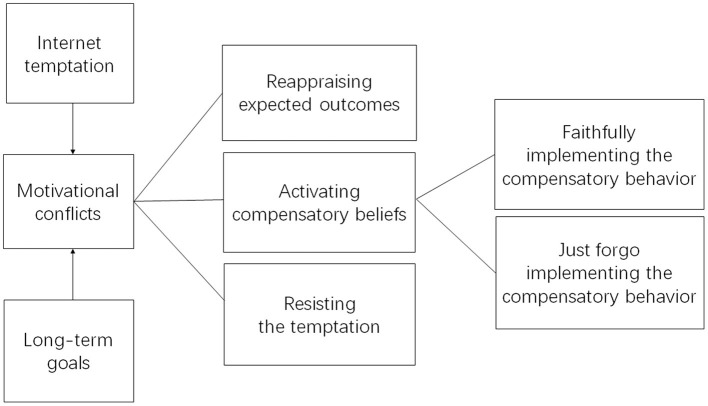
The compensatory belief model for internet gratification behaviors.

After the CB theory was originally put forward to explain health maintenance behaviors, it was applied to research other behaviors such as driving safety, green travel, and smoking (13–19). Following this trend, the present study examines whether the CB theory may also help explain different individuals' decision making when facing with the Internet temptation, as well as their psychology and behavior afterwards. A model based on the CB theory for IG behaviors is shown in Figure 1.

Most of the previous research methods on CB used the survey method with questionnaires (13–19). However, for people who are accustomed to instant gratification, a form of game-based assessment (20) (GBA) may be another viable research method. The GBA refers to the explicit use of information gathered from the game or surrounding activities to ground inferences about players' traits or capabilities–i.e., the behaviors and choices made by players in games can provide researchers with unbiased game data, and through the analysis of these data, individual abilities or characteristics can be evaluated. Among many types of GBA, interactive narratives (IN) are particularly useful because these are stories that allow readers to determine the direction of the plot, often at key decision points (21). By emphasizing on user control, such user-directed storylines in GBA can effectively evaluate behavioral options at critical points. Compared with the traditional questionnaire survey method, the IN-style GBA can effectively and continuously presents the subject with a consequential scenario contingent upon the subject's response to the previous scenario, which can not only more readily simulate the subject's situational reactions in real life, but also fits well with different parts of the CB theory for IG behaviors (as in Figure 1). People who are accustomed to accept the temptation of instant gratification on the Internet generally have the characteristics of sensation seeking (22)–they are keen on seeking novel excitement. Therefore, by providing real-time opportunities to engage in studies with more sensational excitement, the IN-style GBA can increase the target participants' willingness to engage in the study and provide data with higher validity.

In sum, three key exploratory goals were devised for this study. Firstly, we aim to establish an IN-style GBA which can be used to examine individuals' decision making when facing with the temptation of IG. Secondly, we aim to explore whether the CB theory can be applied to the interpretation of IG behaviors. Third, we aim to see if people who have different behavioral choices in front of Internet temptation as predicted by the CB theory would be different in experiencing regrets and other negative emotions at the ending of the narratives. To achieve these goals, we chose college students and organizational employees, two representative groups of the society as they together account for about one thirds of the total population (according to national census data) and common target groups of psychological and public mental health studies, to examine the ecological validity of the CB theory in explaining IG behaviors^1^.

## 2. Methods

### 2.1. Participants

The online smart research platform *Credamo*^2^ and its data collection services were used to design the GBA and recruit participants *via* a random sampling process all across the country. The platform's precise delivery function was used to target specific population groups (college students or organizational employees who are familiar with at least one of the three IG scenarios, see later). Data were excluded if failing at least one attention check^3^ and a total of 1,350 participants were recruited online^4^. Among them, 852 were college students and 498 were organizational employees. Before data analysis, responses from participants who declared that “None of them is familiar to me” at the scenario choosing step in both samples were excluded (36 responses were excluded from the student sample and 16 responses were excluded from the employee sample). Finally, 816 responses were analyzed for college students and 482 responses were analyzed for organizational employees. In the student cohort, there were 266 males and 550 females with a mean age of 21.17 (SD = 2.01, range 15–30); in the employee cohort, there were 249 males and 233 females with a mean age of 31.19 (SD = 7.09, range 18–58). More demographic information on the two samples is shown in Supplementary material (Tables S1A, S1B).

### 2.2. Instruments

The interactive-narrative-style game-based assessment of Internet gratification behavior (IN-GBA-IGB) was presented in the form of an interactive questionnaire, which simulates the behavioral choices an individual takes when facing with the temptation of instant gratification on the Internet. The process of constructing the IN-GBA-IGB was as follows:

Firstly, in the early stage of the research, we interviewed 10 college students and organizational employees, including 5 males (2 students and 3 employees) and 5 females (3 students and 2 employees). The content of the interview was about how participants would respond when facing with the temptation of instant gratification on the Internet. Based on the content of the interview, it was possible to understand the interviewee's choices when facing with the conflicts between the Internet temptation and long-term goals, as well as the beliefs to compensate for them. Through the coding of the interview data, three types of scenarios were determined to be used in the GBA: online gaming, online drama and online shopping. At the same time, three frequent choices of individuals when facing with the conflicts between the Internet temptation and long-term goals were determined: directly accepting the temptation *via* cognitively reappraising expected outcomes, resisting the temptation with resoluteness, and activating some kind of CB before accepting the temptation. In addition, after activating the CB, there were two situations of individual's choices: to faithfully perform the compensatory behavior and to just forgo performing the compensatory behavior. Annotated scripts of the assessment can be found at Supplementary material (Credamo+IN-GBA-IGB-S and Credamo+IN-GBA-IGB-E for college students and organizational employees, respectively).

## 3. Results

The descriptive statistics of the two versions of the IN-GBA-IGB for college students and for organizational employees are shown in Tables 1A, B, respectively. It was found that almost identical percentages of participants chose the online gaming scenario for the student group (36.4%) and the employee group (35.3%), whereas there were more participants choosing online shopping for the employee group (39.6%) than the student group (25.9%), and more participants choosing online drama for the student group (37.7%) than the employee group (25.1%), reflecting a life-style difference between the two groups. In terms of behavioral options in front of the Internet temptation scenario they had chosen, more students directly accepted the temptation (28.4%) than the employees (14.5%), and less students resisted the temptation (32.7%) than the employees (46.7%), possibly reflecting the different maturity of their self-control. However, there were almost identical percentages of participants between the two groups who chose to activate the CB and then accepted the temptation (Students: 38.9%; Employees: 38.8%), reflecting a plausible popularity of the CB phenomenon in IG behavior. Among these participants who activated CB, slightly more employees faithfully performed the compensatory behavior than students (Students: 25.4%; Employees: 28.2%), and slightly more students just forwent performing the compensatory behavior than employees (Students: 13.5%; Employees: 10.6%). For all participants analyzed, more students reported regrets after the ending of the narratives than employees (Students: 29.4%; Employees: 14.1%).

**Table 1A:** The descriptive statistics of GBA-IGB for college students (*N* = 816).

**Item**	**Type**		***n* (%)**
Internet gratification scenarios	Online gaming		297 (36.4)
	Online shopping		211 (25.9)
	Online drama		308 (37.7)
Behavioral options	Cognitively reappraised expected outcomes and accepted the temptation		232 (28.4)
	Resisted the temptation with resoluteness		267 (32.7)
	Activated compensatory beliefs and accepted the temptation	Faithfully performed the compensatory behavior	207 (25.4)
Forwent performing the compensatory behavior		110 (13.5)
Whether to regret	No		576 (70.6)
	Yes		240 (29.4)

**Table 1B:** The descriptive statistics of GBA-IGB for organizational employees (*N* = 482).

**Item**	**Type**		***n* (%)**
Internet gratification scenarios	Online gaming		170 (35.3)
	Online shopping		191 (39.6)
	Online drama		121 (25.1)
Behavioral options	Cognitively reappraised expected outcomes and accepted the temptation		70 (14.5)
	Resisted the temptation with resoluteness		225 (46.7)
	Activated compensatory beliefs and accepted the temptation	Faithfully performed the compensatory behavior	136 (28.2)
		Forwent performing the compensatory behavior	51 (10.6)
Whether to regret	No		414 (85.9)
	Yes		68 (14.1)

A chi-square test was performed to test whether people who chose differently in the narratives would experience regrets at the ending of the narratives, and the results are shown in Tables 2A, B. It was found that for students, those who chose to directly accept the temptation had significantly more participants reporting regrets than those who reported no regrets, and the case was the opposite for employees, suggesting that employees were more willing to “just let it go.” For those who chose to resist the temptation, there were universally large proportion of participants reporting no regrets, confirming the resoluteness of these portion of participants. For those who chose to activate CB and faithfully performed compensatory behaviors, there were also universally large proportion of participants reporting no regrets, whereas for those who chose to activate CB but just forwent performing compensatory behaviors, there were universally significantly more participants reporting regrets than those who reported no regrets. These results suggest that on one hand, the faithful performance of compensatory behavior might have helped participants gain self-efficacy and thus less tendency to feel regretted but the inconsistency between CB and the actually compensatory behavior might be the root of “negative emotional spiral”^5^ when engaging in IG behavior. These hypotheses are supported by an analysis of participants' descriptive mood words immediately after they reported whether to regret. As shown in Tables 3A, B, these regrets were accompanied by a certain degree of other negative emotions, such as feeling lost, sad, guilty, lonely or hopeless. At the same time, many more positive emotions such as delighted, fulfilled, peaceful, happy and contented can be found in participants who resisted the temptation or activated CB and faithfully performed the compensatory behavior.

**Table 2A:** Regrets of different options (college students).

	**Whether to regret**		***χ^2^***	***p***
	**No**	**Yes**		
Cognitively reappraised expected outcomes and accepted the temptation	101^a^	131^b^	234.17	< 0.001
Resisted the temptation with resoluteness	257^a^	10^b^		
Activated compensatory beliefs and faithfully performed the compensatory behavior	174^a^	33^b^		
Activated compensatory beliefs but forwent performing the compensatory behavior	44^a^	66^b^		

If the letters in the superscripts are different, they indicate that there exist significant differences at the level of 0.05 regarding the choice of “Whether to regret”.

**Table 2B:** Regrets of different options (organizational employees).

	**Whether to regret**		***χ^2^***	***p***
	**No**	**Yes**		
Cognitively reappraised expected outcomes and accepted the temptation	49^a^	21^b^	113.92	< 0.001
Resisted the temptation with resoluteness	221^a^	4^b^		
Activated compensatory beliefs and faithfully performed the compensatory behavior	121^a^	15^b^		
Activated compensatory beliefs but forwent performing the compensatory behavior	23^a^	28^a^		

If the letters in the superscripts are different, they indicate that there exist significant differences at the level of 0.05 regarding the of choice of “Whether to regret”.

**Table 3A:** Emotional word coding table (college students).

	**Positive**		**Negative**			
	**Emotional word**	***n (%)***	**Emotional word**	***n (%)***	**Emotional word**	***n (%)***
Cognitively reappraised expected outcomes and accepted the temptation	Delighted	64 (31.1)	Lost	41(19.9)	Helpless	5(2.4)
	Peaceful	17 (8.3)	Regretted	12 (5.8)	Melancholy	3 (1.5)
	Fulfilled	7 (3.4)	Fatigued	8 (3.9)	Empty	2 (1.0)
	Happy	7 (3.4)	Anxious	7 (3.4)	Irritable	1 (0.5)
	Easy	3 (1.5)	Regrettable	7 (3.4)	Frustrated	1(0.5)
	Hopeful	1 (0.5)	Disappointed	7 (3.4)	Pitiful	1 (0.5)
			Guilty	6 (2.9)	Ashamed	1 (0.5)
			Remorseful	5 (2.4)		
	**Positive**		**Negative**			
	**Emotional word**	***n (%)***	**Emotional word**	***n (%)***		
Resisted the temptation with resoluteness	Fulfilled	96 (37.6)	Lost	6 (2.4)		
	Delighted	76 (29.8)	Bored	2 (0.8)		
	Pleased	26 (10.2)	Anxious	1 (0.4)		
	Happy	19 (7.5)	Exhausted	1 (0.4)		
	Peaceful	14 (5.5)	Nervous	1 (0.4)		
	Achievement	5 (2.0)	Worried	1 (0.4)		
	Comfortable	3 (1.2)	Depressed	1 (0.4)		
	Full of energy	1 (0.4)	Busy	1 (0.4)		
	Spirited	1 (0.4)				
	**Positive**		**Negative**			
	**Emotional word**	***n (%)***	**Emotional word**	***n (%)***	**Emotional word**	***n (%)***
Activated compensatory beliefs but forwent performing the compensatory behavior	Delighted	27 (25.2)	Lost	29 (27.1)	Exhausted	2 (1.9)
	Calm	7 (6.5)	Anxious	7 (6.5)	Unhappy	1 (0.9)
	Fulfilled	3 (2.8)	Regretted	6 (5.6)	Pitiful	1 (0.9)
	Happy	2 (1.9)	Disappointed	5 (4.7)	Stressful	1 (0.9)
	Contended	1 (0.9)	Empty	4 (2.4)	Irritated	1 (0.9)
			Struggling	4 (2.4)	Helpless	1 (0.9)
			Confused	2 (1.9)	Busy	1 (0.9)
			Guilty	2 (1.9)		
	**Positive**		**Negative**			
	**Emotional word**	***n (%)***	**Emotional word**	***n (%)***	**Emotional word**	***n (%)***
Activated compensatory beliefs and faithfully performed the compensatory behavior	Delighted	88 (43.3)	Lost	11 (5.4)	Worried	2 (1.0)
	Fulfilled	32 (15.8)	Regretted	6 (3.0)	Uncomfortable	1 (0.5)
	Peaceful	16 (7.9)	Anxious	5 (2.5)	Busy	1 (0.5)
	Happy	13 (6.4)	Exhausted	5 (2.5)	Feel like a dilemma	1 (0.5)
	Contended	10 (4.9)	Hesitant	4 (2.0)	Mediocre	1 (0.5)
			Guilty	3 (1.5)	Remorseful	1 (0.5)
			Empty	2 (1.0)		
			Disappointed	1 (0.5)		

**Table 3B:** Emotional word coding table (organizational employees).

	**Positive**		**Negative**			
	**Emotional word**	***n (%)***	**Emotional word**	***n (%)***	**Emotional word**	***n (%)***
Cognitively reappraised expected outcomes and accepted the temptation	Delighted	23 (35.9)	Lost	8 (12.5)	Worried	1 (1.6)
	Happy	12 (18.8)	Disappointed	3 (4.7)	Confused	1 (1.6)
	Pleased	4 (6.3)	Upset	2 (3.1)	Nervous	1 (1.6)
	Fulfilled	2 (3.1)	Sad	2 (3.1)	Busy	1 (1.6)
	Easy	1 (1.6)	Anxious	1 (1.6)		
	Optimistic	1 (1.6)	Helpless	1 (1.6)		
	**Positive**		**Negative**			
	**Emotional word**	***n (%)***	**Emotional word**	***n (%)***		
Resisted the temptation with resoluteness	Delighted	85 (43.1)	Lost	3 (1.5)		
	Fulfilling	54 (27.4)	Unsuitable	1 (0.5)		
	Happy	23 (11.7)	Stressful	1 (0.5)		
	Hopeful	8 (4.1)	Hesitant	1 (0.5)		
	Confident	5 (2.5)	Confused	1 (0.5)		
	Targeted	4 (2.0)	Struggling	1 (0.5)		
	Calm	4 (2.0)	Busy	1 (0.5)		
	Relaxed	2 (1.0)				
	Firm	2 (1.0)				
	Optimistic	1 (0.5)				
	**Positive**		**Negative**			
	**Emotional word**	***n (%)***	**Emotional word**	***n (%)***		
Activated compensatory beliefs but forwent performing the compensatory behavior	Delighted	17 (34.0)	Lost	11 (22.0)		
	Happy	5 (10.0)	Regretted	3 (6.0)		
	Fulfilled	2 (4.0)	Guilty	2 (4.0)		
	Relaxed	2 (4.0)	Upset	1 (2.0)		
	Easy	1 (2.0)	Disappointed	1 (2.0)		
	Bright	1 (2.0)	Confused	1 (2.0)		
	Comfortable	1 (2.0)	Remorseful	1 (2.0)		
			Busy	1 (2.0)		
	**Positive**		**Negative**			
	**Emotional word**	***n (%)***	**Emotional word**	***n (%)***		
Activated compensatory beliefs and faithfully performed the compensatory behavior	Cheerful	59 (42.1)	Lost	6 (4.3)		
	Fulfilled	24 (17.1)	Regretted	5 (3.6)		
	Happy	22 (15.7)	Sad	2 (1.4)		
	Contended	6 (4.3)	Guilty	1 (0.7)		
	Relaxed	4 (2.9)	Confused	1 (0.7)		
	Worthwhile	3 (2.1)	Unhappy	1 (0.7)		
	Just-as-usual	3 (2.1)	Tired	1 (0.7)		
	Calm	2 (1.4)	Bored	1 (0.7)		

## 2.3. Procedure

After the participants answered demographic variable questions, the IN-GBA-IGB was presented. The procedure consists of six steps as follows:

1. After answering demographic questions, the participants could choose among three representative IG scenarios (see above), or they could choose “none of them was familiar to me.” If the participants chose “none of them was familiar to me,” the assessment would end and the participants were thanked for their participation.
2. After one of the three scenarios was selected, the game plot of the selected scenario would be displayed, and the participants would be informed of their current long-term goals depending on their identity (students or employees), which would cause cognitive conflicts between the temptation of instantly gratifying on the Internet and rejecting the temptation for achieving long-term goals. After the participants read the game plot, they were asked to repeat the content in the game plot in order to confirm that the participant had a full understanding of their game plot.
3. Then, the participants were presented with a new page which the temptation of the chosen type of scenario was imminent, and the participants could choose among three monologs that represented their different choices: directly accepting the temptation *via* cognitively reappraising expected outcomes, resisting the temptation with resoluteness, and activating a corresponding kind of CB before accepting the temptation. There were five logically-coherent dialogues or monologs waiting to be consecutively presented to the participants in order to “lure” them to accept the temptation (e.g., in the online gaming scenario section: “You are getting ready to study on one weekend morning. At this time, a close friend asks you to log into the game, and here's what you will do”; or in the online shopping scenario section: “It doesn't take much of your time, and it's a team competition. You can join in to play or not. We can help you. Just come and do the task when you have time.”). If the participants chose to resist the temptation (e.g., in the online gaming scenario section: “No, I'm getting ready to study”; or in the online shopping scenario section: “No, I think I won't participate in the program this year.”), the next one would appear (e.g., in the online gaming scenario section: “Why do you still study on weekends? Are you not tired yet after working for 5 days? Take a good rest, you have to balance work and rest”; or in the online shopping scenario section: “It doesn't take much of your time, and it's a team competition. You can join in to play or not. We can help you. Just come and do the task when you have time”), until the participant directly accepts the temptation (e.g., in the Online gaming scenario section: “Okay, let's play”; or in the Online shopping scenario section: “Okay, anyway, I've been waiting to buy something recently, so I can make some deal.”) or activated the CB (e.g., in the Online gaming scenario section: “ I'll just play games for a while, and then study for two more hours in the afternoon to make up for it. ”; or in the Online shopping scenario section: “Let me join them first, and I'll... ” is chosen, jump to: “Then you happily join the program…”).
4. Those participants who chose the option of activating the CB would then be presented with the next plot describing that they had enjoyed the Internet usage for long and were then presented with the opportunity to perform the compensatory behavior (e.g., in the Online gaming scenario section: “This kind of opportunity can come at any time. Next time, I'll take the initiative to ask him (her) to play”; or in the Online shopping scenario section: “I have been satisfied just now. Let me continue to study more to make up for the time.”) despite for that they were already mentally tired and some other temptation came up, or that they can still choose to forwent performing the compensatory behavior (e.g., in the Online gaming scenario section: “This kind of opportunity doesn't come often. Let's play with him (her) for a while”; or in the Online shopping scenario section: “I'm tired from work during the day, just go to rest.”).
5. Different story ends would ensue depending on the participants' choices, and finally the participants' descriptive moods and whether they felt regretted for their choices were collected for analysis.
6. At the end of the GBA, the participants were provided with an explanatory session for the study and ways of psychological assistance if needed. All participants were compensated *via* their *Credamo* accounts for their participation.

The protocols of the current study were approved by the Institutional Review Board of School of Psychology, Fujian Normal University (Approval No. 2020090402).

## 4. Discussion

The current study applies an IN-GBA to demonstrate a case in using the CB theory to explain the cause of IG behaviors, which can be regarded as an extension of the application areas of the CB theory. In social and behavioral sciences, a theory is constructed to explain certain phenomenon in human behavior, which is bounded by the type of population it concerns, the context where the theory is at play and the methods used to test the hypothesis predicted by the theory (23, 24). In this regard, when expanding a theory into new frontiers, one has to make sure that similar phenomenon does exist in the new area, and people's behavioral sequences there match well with the explanation of the theory. This is what we aimed for in this study. However, due to the long-debated discrepancy between attitudes and behavior, traditional methods based on retrospective introspection may not be the best choice to reflect what people are actually behaving in situations such as the Internet temptation (25). For example, the study of Jia et al. (26) found that those subjects with good self-reported self-control ability were the most indebted in the situational behavior test; based on 106 effect sizes, Parry et al. (27) also discovered that self-reported digital media use were rarely an accurate reflection of logged media use and the correlation were even weaker for problematic media use. Therefore, in order to more accurately assess the existence of IG behavior predicted by the CB theory, a methodology that can engage participants in real-time scenarios and provides interactive feedback to participants' responses may be the better choice. Following this logic, we adopted the IN-style GBA, having participants select the IG scenario they were most familiar with and experiencing interactively in the corresponding story. When building the GBA, the storylines was designed according to the structure and predictions of the CB model. Specifically, because the CB model predicts three different possible choices an individual can make when facing with the IG temptation, and two further choices if CB activation was the choice at the first step, the interactive narratives were adopted so that the logic flow was more in line with the CB model and also fits well with participants of different characteristics, enhancing the ecological validity of the assessment.

To be more specific, when facing with the conflicts between long-term goals and the temptation of instant gratification on the Internet, the percentages of participants who chose to directly accept the temptation or accepting the temptation after activating the CB are collectively higher than those who consistently chose to refuse the temptation, suggesting that people are more inclined to give higher weights to events that can instantly benefit them, that is, to accept the IG, which is consistent with previous study findings (5, 8–12). Notably, according to our qualitative interviews in the preparation phase of the IN-GBA, college students and organizational employees are at different stages of their lives (age included) with generally different socioeconomic basis, having different long-term goals to strive for, and importantly having different lifestyles (especially how they spend weekdays vs. weekends). These differences would affect the type of Internet temptation they are most familiar with, the scenario that put them into conflicts between striving for long-term goals and engaging in instant Internet gratification behavior, and the specific content of the compensatory beliefs they would be more likely to activate. Our data also demonstrate these differences as a phenomenon, but importantly the proportion of sample who choose to activate compensatory beliefs before accepting the Internet temptation were similar across the student group and the employee group (close to 40%), suggesting that activating compensatory beliefs may be a strategy to deal with cognitive conflicts between striving for long-term goals and engaging in instant Internet gratification behavior for a certain part of the population.

However, if they fail to perform compensatory behaviors later, the outcome may further cause cognitive conflicts to resume and exacerbate with negative emotions. These discomforts will continue until compensatory behavior is performed or other methods of coping with cognitive conflicts are implemented (5). If an individual continues to fall into the malicious cycle of activating the CB and accepting the IG but forgoing performing the compensatory behavior, not only may the behaviors evolve into Internet addiction, but the individual may also develop symptoms of depression (28, 29). Therefore, public mental health practitioners may need to pay more attention to this cohort of the population, devise guidance and tools from the standpoint of preventative medicine, for the purpose of reducing the incidence of Internet addiction.

Despite that GBA has attracted a lot of attention in the educational/psychological assessment (30–34) and human resource management area (35–37), it has not become a standard tool in the public health domain. The IN-style GBA used in the present study was only a primer of what such assessment can be like. Due to the differences between the GBA and traditional evaluation methods, the reliability and validity of this method may not easily be reflected through inferential statistical analysis. Future research can design a way to present the GBA that is more similar to video games, collecting systematic game log data that can be used for systematic analysis, which would allow formal and quantitative testing of hypothesis derived from the CB theory and the like. After all, the GBA can be especially useful during a pandemic period with strict public health policies and become a powerful tool in the era of the metaverse in the future.

## Data availability statement

The original contributions presented in the study are included in the article/Supplementary material, further inquiries can be directed to the corresponding author.

## Ethics statement

The studies involving human participants were reviewed and approved by the Institutional Review Board of School of Psychology, Fujian Normal University (Approval No. 2020090402). The patients/participants provided their written informed consent to participate in this study.

## Author contributions

BY and YS conceived of the present project, designed the study, and collected the data. YS analyzed the data and wrote the first draft of the manuscript. BY critically revised the manuscript. Both authors approved the final version of the manuscript.

## Footnotes

^1^Internet gratification behaviors are rather common among different groups of people, but different groups of people have different goals, daily schedules and lifestyles and hence may exhibit different scenarios for the same construct. For example, college students and organizational employees may treat weekdays and weekends differently because of their different time structure, resulting in different content of the compensatory beliefs. Therefore, in some cases it is better to have different versions of the assessment for different groups. As for why we only chose college students and organizational employees here: on one hand these were two largest demographic groups in the subject pool of *Credamo*, on the other hand these two groups were living in different contexts and thus were good for comparison to test for the ecological validity of the CB phenomenon in IG behavior.

^2^A powerful, large-scale and reliable smart research platform that provides both online research design and data collection services in both the Chinese and English languages (https://www.credamo.com). It has a subject pool of more than 2.8 million participants and is nationally representative all across different age, gender, income, education and career fields.

^3^Three attention checks were set up in the interactive-narrative questionnaire. Firstly, in the demographic information section, there was a question about the participant's profession (“Please select your profession”), data selected for “Students” in the organizational employee version and data selected other than “Students” selected in the college student version were excluded. Secondly, after the participant were instructed to read the scenario corresponding to his/her scenario choice, there was a fill-in-the-blanks question asking the participant to retell the content of the scenario as much as possible to confirm that he/she had read and understood the scenario carefully. The participants' responses to this question were evaluated for quality, and data showing that the participants did not understand the scenario were excluded. Thirdly, at the end of the questionnaire, there was another fill-in-the-blanks question asking the participants to freely express his/her feeling at the moment, and data with overly perfunctory answers were excluded.

^4^According to the above criteria, we received 925 responses but excluded 73 responses for the sample of college students, and received 558 responses but excluded 60 responses for the sample of organizational employees, resulting in 852 responses for college students and 498 responses for organizational employees and with response quality ensured.

^5^This is a term we coined to describe the process during which negative emotions elicited after forgoing compensatory behavior lead to low self-efficacy, and expected self-efficacy leads to further compensatory beliefs without performing compensatory behavior, which then leads to deeper negative emotions afterwards.
