# Supplementary material for: Compensatory beliefs in the internet gratification behavior: A study of game-based assessment
Source: Front Public Health. 2023 Jan 24;11:997108. doi: 10.3389/fpubh.2023.997108 (PMC9902763; doi:10.3389/fpubh.2023.997108)
Supplement: Supplementary file 1 [file Table_1.docx]

**Table S1A** Demographic Information (College Students)

| Item | Category | Percentage (%) | |  |
| --- | --- | --- | --- | --- |
| Gender | Male | 32.51 | |  |
|  | Female | 67.49 | |  |
|  |  |  | |  |
| Age | 18-22 | | 79.52 | |
|  | 23-26 | | 18.57 | |
|  | 27-30 | | 1.43 | |
|  | >30 | | 0.48 | |
|  |  | |  | |
| Grade | Freshmen | 21.83 | |  |
|  | Sophomore | 27.93 | |  |
|  | Junior | 24.41 | |  |
|  | Senior | 13.85 | |  |
|  | Master’s Students | 10.56 | |  |
|  | Doctoral Students | 1.41 | |  |
|  |  |  | |  |
| Majors | Humanities  Engineering  Arts | 19.60  36.03  4.11 | |  |
|  | Sports | 0.70 | |  |
|  | Medicine | 6.69 | |  |
|  | Social Sciences | 16.43 | |  |
|  | Natural Sciences | 3.87 | |  |
|  | Others | 12.56 | |  |
|  |  |  | |  |
| Whether or not student leaders | Yes | 53.76 | |  |
|  | No | 46.24 | |  |

**Table S1B** Demographic Information (Organizational Employees)

| Item | Category | Percentage (%) |
| --- | --- | --- |
| Gender | Male | 51.61 |
|  | Female | 48.39 |
|  |  |  |
| Age | ≤20 | 0.99 |
|  | 21-30 | 51.48 |
|  | 31-40 | 36.96 |
|  | 41-50 | 5.94 |
|  | >50 | 4.62 |
|  |  |  |
| Education | Doctoral degree | 1.20 |
|  | Master’s degree | 9.64 |
|  | Bachelor’s degree | 67.47 |
|  | Associate degree | 13.86 |
|  | High school degree | 6.63 |
|  | Junior high school degree or below | 1.20 |
|  |  |  |
| Years of working experiences | Less than 3 years | 18.88 |
|  | 3-5 years | 34.34 |
|  | 6-10 years | 31.12 |
|  | 11 years and above | 15.66 |
| Job title | Basic-level management/junior technician/section level | 73.69 |
|  | Middle-level management/intermediate technician/division level | 20.08 |
|  | Senior management/senior technician/bureau level | 6.22 |
| Income | 15,000 yuan and above | 8.70 |
|  | 8001-15000 yuan | 29.43 |
|  | 6501-8000 yuan | 26.42 |
|  | 5001-6500 yuan | 16.05 |
|  | 3501-5000 yuan | 14.05 |
|  | 3000 yuan and below | 5.35 |
