# Supplementary material for: Compensatory beliefs in the internet gratification behavior: A study of game-based assessment
Source: Front Public Health. 2023 Jan 24;11:997108. doi: 10.3389/fpubh.2023.997108 (PMC9902763; doi:10.3389/fpubh.2023.997108)
Supplement: Supplementary file 3 [file Presentation_2.pdf]

IN-GBA-IGB-E

Hello! Thank you for participating in this game-based survey. Your participation is anonymous, and the results of the survey will only be used for research per your permission. Please read the scenarios and options/questions carefully and answer them truthfully. In addition to your basic information, the main part of the survey will take you about five minutes.

Note: This game-based survey is for organizational employees only. If you do not identify yourself as an organizational employee, the survey will end after a few demographic questions.

Demographic information - Section starts

Your gender: [Choose one answer below]

- ○ Male
- ○ Female
- ○ Prefer not to say

Your age: [Slide to the most appropriate place]

	1-100
Age	

Please select your profession: [Choose one answer below]

- ○ Students
- ○ Employees at a state-owned enterprise
- ○ Employees at a public-sector organization
- ○ Employees at a private-sector organization
- ○ Employees at a foreign-owned organization
- ○ Civil servant
- ○ Self-employed or freelancer
- ○ Retired
- ○ Others

What is your educational background? [Choose one answer below]

- ○ Doctoral degree
- ○ Master’s degree
- ○ Bachelor’s degree
- ○ Associate degree
- ○ High school degree
- ○ Junior high school degree or below

What was your major during school? [Choose one answer below]

- ○ Humanities
- ○ Engineering
- ○ Arts
- ○ Sports
- ○ Medicine

- Social Sciences
- Natural Sciences
- Others

Your working years: [Choose one answer below]

- 3 years or less
- 3-5 years
- 6-10 years
- 11 years and above

Your job position: [Choose one answer below]

- General staff
- Basic-level management/junior technical staff/section level
- Middle-level management/intermediate technical staff/division level
- Senior-level management / senior technical staff /department level

Demographic information - Section ends

Scenario choice - Section starts

Which of the following Internet activities gives you the most satisfaction? [Choose one answer below]

- Online gaming
- Online shopping
- Online drama
- None of them is familiar to me

Scenario choice - Section ends

Online gaming scenario - Section starts (only if "Online gaming" is chosen in the "Scenario choice" section)

Instructions: Please read the following text carefully, and **prepare paper and pen to keep notes for retelling**.

You are a skilled player of "Game for Peace"/ "Honor of Kings". You often play games with your classmates and friends. You not only experience the fun of the game, but also gain friendship through playing these games.

Your company recently has a plan for employee training and development at company expenses, but the number of openings is limited, and you need to pass the assessment before you can get the opportunity. There will be opportunities for promotion and salary increase after completing the training. As you get used to the job, you start thinking about the future.

Now you have decided to use your time outside of work to study and work hard to get this opportunity.

However, on the one hand, many colleagues also want to get this opportunity for further study, and the

competition is a bit fierce. At the same time, the preparation process requires a lot of time and energy, and you only have time to study after getting off work each day and the weekends, which is limited; on the other hand, it seems a little unrealistic to suddenly give up all the happiness gained in the game playing.

Please retell the content of the scenario as much as possible to confirm that you have read and understood it carefully. [Fill in the blanks here]

____

Now, please review the scenario you just retold and make the following choice according to your thoughts and feelings.

You are getting ready to study one weekend morning. At this time, a friend who is usually close to you asks you to log in to the game, and then you will: [Choose one answer below]

○ "No, I'm getting ready to study."
○ "Okay, just two rounds of play."
○ "I'll just play games for a while, and then study for two more hours in the afternoon to make up for it."

Condition："No, I'm getting ready to study." is chosen, jump to: "Why do you still study on weekends? ..."
Condition："Okay, just two rounds of play." is chosen, jump to: "You guys played this game until noon..."
Condition："I'll just play games for a while..." is chosen, jump to: "So you played games with your friends..."

"Why do you still study on weekends? Are you not tired yet after working for five days? Take a good rest, you have to balance work and rest." [Choose one answer below]

○ "No-no, morning is a good time to study for me."
○ "Okay, let's play."
○ "I'll just play for a while, and then study for two more hours in the afternoon to make up for it."

Condition："No-no, morning is a good time to study... " is chosen, jump to: "It's fine to play for a while..."
Condition："Okay, let's play." is chosen, jump to: "You guys played this game until noon..."
Condition："I'll just play for a while, and then... " is chosen, jump to: "So you played games with your..."

"It's fine to play for a while, just one round, it won't take much time." [Choose one answer below]

○ "No, it will take a long time to play just one round. We may not be able to resist playing the second and third rounds."
○ "Alright, just one round."
○ "I'll just play games for a while, and then study for two more hours in the afternoon to make up for it."

Condition："No, it will take a long time to play..." is chosen, jump to: "Hey come on, we have a game..."
Condition："Alright, just one round." is chosen, jump to: "You guys played this game until noon..."
Condition："I'll just play games for a while..." is chosen, jump to: "So you played games with your friend... "

"Hey come on, we have a game master to help us!" [Choose one answer below]

○ "Awesome, I'll definitely come next time."

- "Then let's play a few rounds."
- "I'll just play games for a while, and then study for two more hours in the afternoon to make up for it."

Condition："Awesome, I’ll definitely come next..." is chosen, jump to: "Oh, you're such a disappoint..."
Condition："Then let's play a few rounds." is chosen, jump to: "You guys played this game until noon... "
Condition："I'll just play games for a while..." is chosen, jump to: "So you played games with your friend…"

"Oh, you're such a disappointment, go to study then. I'll call you next time." [Choose one answer below]

- "Sorry, I really don't want to play now. I'll invite you to play next time when I'm free."
- "My plans for this week are almost done, now let me just relax a bit."
- "I'll play games for a while, and then study for two more hours in the afternoon to make up for it."

Condition："Sorry, I really don't want to play..." is chosen, jump to: "You spent an ordinary weekend for..."
Condition："My plans for this week are almost..." is chosen, jump to: "You guys played this game until..."
Condition："I'll play games for a while..." is chosen, jump to: "So you played games with your friends..."

So you played games with your friends until lunchtime. Now you decide to study two more hours from the afternoon to the evening to make up for the time you lost in the morning. However, in the afternoon the phone rang again, this time someone you have a feeling on sent you a message, asking you to play the game online, then you will: [Choose one answer below]

- "This kind of opportunity doesn't come often. Let's play with him (her) for a while."
- "This kind of opportunity can come at any time. Next time, I’ll take the initiative to ask him (her) to play."

Condition："This kind of opportunity doesn't come..." is chosen, jump to: "You guys played this game until...
Condition："This kind of opportunity can come..." is chosen, jump to: "You planned to study hard all day..."

You guys played this game until noon. You regained the feeling you had before while playing this online game with your friends. You are in a happy mood, but you have already left things behind on your study plan.

You spent an ordinary weekend for executing your study plan, and you watched yourself approaching your goal little by little, and it felt worthwhile in your heart, because you know how excited you will be to see your future success.

You planned to study hard all day, but you also spent time playing online games with your friends in the morning. After all, you had a great time, and because there was not much lost in study and entertainment, you feel that the day was richer.

In this scenario, did you regret making the choice? [Choose one answer below]

- ○ Yes
- ○ No

Why? [Fill in the blanks here]

____

Please use one word to describe how you feel (e.g. sad, happy, hopeless) when you choose to live your day this way: [Fill in the blanks here]

____

Online gaming scenario-Section ends

Online shopping scenario-Section starts (only if “Online shopping” is chosen in the “Scenario choice” section)

Instruction: Please read the following scenario carefully, and **prepare paper and pen to keep notes for retelling.**

You spend a lot of money on online shopping every month, and in order to make sure you are spending them on the right place, you also spend time reading various reviews and comparing various products. You recently spotted a nice suit but couldn't bear to buy it for now, so you add it to your e-shopping cart.

Your company recently has a plan for employee training and development at company expenses, but the number of openings is limited, and you need to pass the assessment before you can get the opportunity. There will be opportunities for promotion and salary increase after completing the training. As you get used to the job, you start thinking about the future.

Now you have decided to use your time outside of work to study and work hard to get this opportunity.

However, on the one hand, many colleagues also want to get this opportunity for further study, and the competition is a bit fierce. At the same time, the preparation process requires a lot of time and energy, and you only have time to study after getting off work each day and the weekends, which is limited; on the other hand, the fun of online shopping makes you accustomed to enjoying it, and it seems a little unrealistic to give it up suddenly.

Please retell the content of the scenario as much as possible to confirm that you have read and understood it carefully. [Fill in the blanks here]

____

Now, please review the scenario you just retold and make the following choice according to your thoughts and feelings.

The e-commerce shopping festival is coming soon. Some e-commerce websites have launched marketing and sales programs like in previous years. You can get cash back by teaming up with friends to do certain activity tasks. In previous years, you teamed up with good friends and actively participated in the

e-commerce shopping festival activities, and got a lot of cash back. Today, your good friend also sent an invitation message, asking you to join his/her team to "divide" the cashback earned together. Here's what you'll do: [Choose one answer below]

- "No, I have to spend time studying."
- "It's only once a year anyway, let’s go for it.”
- "Let me join them first, but I’ll study for two more hours afterwards every day."

Condition："No, I have to spend time studying." is chosen, jump to: "It doesn't take much of your time..."
Condition："It's only once a year anyway..." is chosen, jump to: "At 0:00 on 11.11, you excitedly snapped..."
Condition："Let me join them first, and I..." is chosen, jump to: "Then you happily join the program with..."

"It doesn't take much of your time, and it's a team competition. You can join in to play or not. We can help you. Just come and do the task when you have time." [Choose one answer below]

- "Oh no, I won't be able to resist the temptation after I join it."
- "Okay, anyway, I've been waiting to buy something recently, so I can make some deal."
- "Let me join them first, but I’ll study for two more hours afterwards every day."

Condition："Oh no, I won't be able to resist..." is chosen, jump to: "This year's program is more interesting..."
Condition："Okay, anyway, I've been waiting..." is chosen, jump to: "At 0:00 on 11.11, you excitedly..."
Condition："Let me join them first, and I..." is chosen, jump to: "Then you happily join the program with..."

"This year's program is more interesting than last year, and the reward looks better than last year." [Choose one answer below]

- "No, I think I won't participate in the program this year."
- "Okay, let's just do it again this year."
- "Let me join them first, but I’ll study for two more hours afterwards every day."

Condition："No, I think I won't participate in..." is chosen, jump to: "Oh, just spend a little time playing..."
Condition："Okay, let's just do it again this year..." is chosen, jump to: "At 0:00 on 11.11, you excitedly..."
Condition："Let me join them first, and I..." is chosen, jump to: "Then you happily join the program with..."

"Oh, just spend a little time playing - you can be happy and also make money - it's better than playing games without money." [Choose one answer below]

- "No, I won't be able to resist the temptation after I join."
- "Okay, anyway, I've been waiting to buy something recently, so I can find some deal."
- "Let me join the activity first, but I’ll study for two more hours afterwards every day."

Condition："No, I won't be able to resist..." is chosen, jump to: "Many friends sent messages asking you..."
Condition："Okay, anyway, I've been waiting..." is chosen, jump to: "At 0:00 on 11.11, you excitedly..."
Condition："Let me join the activity first. I’ll..." is chosen, jump to: "Then you happily join the program..."

Many friends sent messages asking you to help them earn cashback, and you clicked in too. You seem a little out of touch when so many of your friends are attending popular e-commerce programs. Now you will: [Choose one answer below]

○ "Anyway, those programs are held once a year. I won’t join them this year, but I can join them next year."

○ "Anyway, those programs are held once a year. It doesn't take much time to join the team of good friends to participate in some programs."

○ "Let me join the activity first, but I’ll study for two more hours afterwards every day."

Condition："Anyway, those programs are held once..." is chosen, jump to: "You have spent an ordinary...
Condition："Anyway, those programs are held once..." is chosen, jump to: "At 0:00 on 11.11, you excitedly...
Condition："Let me join the activity first, I’ll..." is chosen, jump to: "Then you happily join the program...

Then you happily join the program with your good friends. But after you finish all the tasks the program asks you to do, it has already taken more than an hour, and it will not be long before it is time to rest, and then you will: [Choose one answer below]

○ "I'm tired from work during the day, just go to rest."

○ "I have been satisfied just now. Let me continue to study more to make up for the time."

Condition："I'm tired from work during the day..." is chosen, jump to: "At 0:00 on 11.11, you excitedly... "
Condition："I have been satisfied just now. Let me..." is chosen, jump to: "This year's e-commerce shop... "

At 0:00 on 11.11, you excitedly snapped up the things you wanted to buy for a long time, and got a discount after you worked hard to participate in those e-commerce activities. Although the discount is not much, you are still very happy, but your studies have been delayed.

You have spent an ordinary day in your studies, and it feels worthwhile to watch yourself approaching a great goal little by little. Because you can imagine how excited you will be when you succeed in the future.

This year's e-commerce shopping festival is happier than in previous years, because by teaming up with your good friends to participate in the activity and also study more to make up for the time, few things have been left behind. You feel that this period of time has been very fulfilling.

In this scenario, did you regret making this choice? [Choose one answer below]

○ Yes

○ No

Why？ [Fill in the blanks here]

____

Please use one word to describe how you feel (e.g. sad, happy, hopeless) when you choose to live your day this way: [Fill in the blanks here]

____

Online shopping scenario-Section ends

Online drama scenario - Section starts (only if “Online drama” is chosen in the “Scenario choice” section)

Instructions: Please read the following text carefully, and **prepare paper and pen to keep notes for retelling**.

You are a master of online drama watching. You are members of various video sites, and you know various ways to watch them. When you are free, you always spend them happily watching online dramas. You are accustomed to following several pieces of drama at the same time, and after watching them, you will also make comments on the forum or discuss them with friends. You take them seriously.

Your company recently has a plan for employees to study at public expense, but the number of places is limited, and you need to pass the assessment before you can get the opportunity. There will be opportunities for promotion and salary increase after completing the training. As you get used to the job, you start thinking about the future.

Now you have decided to use your time outside of work to study and work hard to get this opportunity.

However, on the one hand, many colleagues also want to get this opportunity for further study, and the competition is a bit big. At the same time, the preparation process requires a lot of time and energy, and you only have time to study after getting off work and weekends, which is very limited; on the other hand, those dramas you are following is still being updated, and it seems a little unrealistic for you to suddenly give it up.

- -

Please retell the content of the scenario as much as possible to confirm that you have read and understood it carefully. [Fill in the blanks here]

____

Now, please review the scenario you just retold and make the following choice according to your thoughts and feelings.

On Thursday night, you came home from work and sat in your chair thinking, you've been working for so long, and thus it’s better to rest early tonight. Then you will: [Choose one answer below]

○ "I'll continue studying for a while after taking a bath, and go to bed when I'm sleepy."
○ "I'm so tired. I'll just watch the drama and then go to bed tonight."
○ "I'll watch the drama tonight to relax, but I'll spend more time to make up for the study tomorrow night."

Condition： "I'll continue studying for a while..." is chosen, jump to: "At this point you remember that..."
Condition： "I'm so tired. I just watch the drama..." is chosen, jump to: "You watched the new episodes..."
Condition： "I'll watch the drama tonight to relax..." is chosen, jump to: "You went to bed after watching..."

- -

At this point you remember that today is the time for the update of the episode you are following, and then you will: [Choose one answer below]

- "I'm going to bed soon tonight, so just study some more."
- "I'm going to bed soon tonight, so just watch that episode."
- "I'll watch the drama tonight to relax, but I'll spend more time to make up for the study tomorrow night."

Condition："I'm going to bed soon tonight, so just study..." is chosen, jump to: "So I can't study, let me..."
Condition："I'm going to bed soon tonight, so just..." is chosen, jump to: "You watched the new episodes..."
Condition："I'll watch the drama tonight to relax..." is chosen, jump to: "You went to bed after watching..."

"So I can't study, let me finish the show first, just watch one episode." [Choose one answer below]

- "So I can't study, let me finish the show first, just watch one episode."
- "I'll study first, the plot will not develop much in one episode, let them release a few more episodes and then I'll watch it."
- "I'll watch the drama tonight to relax, but I'll spend more time to make up for the study tomorrow night."

Condition："I'll study first, the plot will not..." is chosen, jump to: "So you go to study for a while. You…"
Condition："So I can't study, let me finish..." is chosen, jump to: "You watched the new episodes until…"
Condition："I'll watch the drama tonight to relax..." is chosen, jump to: "You went to bed after watching... "

So you go to study for a while. You get distracted by unconsciously thinking about the plot, and then you: [Choose one answer below]

- "Take a break and continue studying. My study task tonight is almost completed, and I can wait for more updates of the drama before watching them."
- "In this way, my learning efficiency is not very good. Let me watch the drama first. It's just one episode."
- "I'll watch the drama tonight to relax, and I'll spend more time to make up for the study tomorrow night."

Condition："Take a break and continue studying, my..." is chosen, jump to: "You resisted the urge to watch..."
Condition："In this way, my learning efficiency..." is chosen, jump to: "You watched the new episodes..."
Condition："I'll watch the drama tonight to relax..." is chosen, jump to: "You went to bed after watching..."

You resisted the urge to watch the show, and went to the forums to see what people were talking about this episode, and found that this week's updated episode plot was very appealing to you, and you saw a little spoiler, which made you have the urge to watch it , then you would: [Choose one answer below]

- "I'll go to watch it, there's not much time left today."
- "I'll keep learning because there's not much time left today."
- "I'll watch the drama tonight to relax, and I'll spend more time to make up for the study tomorrow night."

Condition："I'll go to watch it, there's not much..." is chosen, jump to: "You watched the new episodes..."
Condition："I'll keep learning because there's..." is chosen, jump to: "You spent an ordinary weekend for..."

Condition："I'll watch the drama tonight to relax..." is chosen, jump to: "You went to bed after watching... "

You went to bed after watching the new episode. The next day, Friday, when you come home at night and learn that another show that you're following has also been updated, you'll: [Choose one answer below]

- ○ "Watch the episode. I still have two days to study hard on weekends."
- ○ "Study. Wait until the weekend when my study tasks are completed and then watch them."

Condition："Watch the episode. I still have two day..." is chosen, jump to: "You watched the new episodes ...
Condition："Study. Wait until the weekend when my..." is chosen, jump to: "This evening, you have more...

You watched the new episodes until going to bed. The plot really lived up to your expectations, and you are in a good mood, feeling that the fatigue of studying all day has been reduced a lot. But the study plan was delayed.

You spent an ordinary weekend for your study plan, and you watched yourself approaching your goal little by little, and it felt worthwhile in your heart, because you know how excited you will be to see your future success.

This evening, you have more time to study than usual. After studying all day during the day, you feel a little more tired in the middle of the night, but you are very happy. Because of the delicate balance between studying and having fun, you feel that the day is richer than it was yesterday.

In this scenario, did you regret making this choice? [Choose one answer below]

- ○ Yes
- ○ No

Why？ [Fill in the blanks here]

____

Please use one word to describe how you feel (e.g. sad, happy, hopeless) when you choose to live your day this way: [Fill in the blanks here]

____

Online drama scenario - Section ends

Concluding remarks - Section starts

The survey is over. Please tell me how you feel now: [Fill in the blanks here]

____

Concluding remarks - Section ends
